# The Effect of Light Intensity, Sensor Height, and Spectral Pre-Processing Methods When Using NIR Spectroscopy to Identify Different Allergen-Containing Powdered Foods

**DOI:** 10.3390/s20010230

**Published:** 2019-12-31

**Authors:** Ahmed Rady, Joel Fischer, Stuart Reeves, Brian Logan, Nicholas James Watson

**Affiliations:** 1 Food, Water, Waste Research Group, Faculty of Engineering, University of Nottingham, Nottingham NG7 2RD, UK; ezzamr@exmail.nottingham.ac.uk; 2 Mixed Reality Laboratory, School of Computer Science, University of Nottingham, Nottingham NG8 1BB, UK; pszjf1@exmail.nottingham.ac.uk (J.F.); pszsr@exmail.nottingham.ac.uk (S.R.); 3 Agents Laboratory, School of Computer Science, University of Nottingham, Nottingham NG8 1BB, UK; pszbsl@exmail.nottingham.ac.uk

**Keywords:** NIR spectroscopy, machine learning, allergen detection, powdered food, industry 4.0, digital manufacturing

## Abstract

Food allergens present a significant health risk to the human population, so their presence must be monitored and controlled within food production environments. This is especially important for powdered food, which can contain nearly all known food allergens. Manufacturing is experiencing the fourth industrial revolution (Industry 4.0), which is the use of digital technologies, such as sensors, Internet of Things (IoT), artificial intelligence, and cloud computing, to improve the productivity, efficiency, and safety of manufacturing processes. This work studied the potential of small low-cost sensors and machine learning to identify different powdered foods which naturally contain allergens. The research utilised a near-infrared (NIR) sensor and measurements were performed on over 50 different powdered food materials. This work focussed on several measurement and data processing parameters, which must be determined when using these sensors. These included sensor light intensity, height between sensor and food sample, and the most suitable spectra pre-processing method. It was found that the K-nearest neighbour and linear discriminant analysis machine learning methods had the highest classification prediction accuracy for identifying samples containing allergens of all methods studied. The height between the sensor and the sample had a greater effect than the sensor light intensity and the classification models performed much better when the sensor was positioned closer to the sample with the highest light intensity. The spectra pre-processing methods, which had the largest positive impact on the classification prediction accuracy, were the standard normal variate (SNV) and multiplicative scattering correction (MSC) methods. It was found that with the optimal combination of sensor height, light intensity, and spectra pre-processing, a classification prediction accuracy of 100% could be achieved, making the technique suitable for use within production environments.

## 1. Introduction

### 1.1. Role of Powdered Foods in the Food Industry

Powdered foods represent a significant share of the modern human diet, mostly as ingredients in food products or cooked meals. Powdered food materials can be baked (e.g., cakes), used as seasoning (e.g., spices), or added to liquids to produce drinks (e.g., powdered milk). Powdered foods can even be made from basic chemicals, such as water and CO_2_ [1], and are essential for future endeavours, such as space exploration [2]. Powders can be defined as, “particulate solid state materials containing discrete particles of size ranging from nanometres to millimetres” [3]. The properties of food powders drastically affect the final product quality and consequently the consumer appreciation. In addition, the primary properties of powders, such as shape and density, have significant effects on product performance, such as rehydration rates of powdered drinks [4]. Therefore, it is important to monitor and measure the primary properties of powdered foods to ensure the final food products have the required performance.

### 1.2. Powder Food Allergens and Digital Technologies in the Food Industry

In addition to monitoring the powdered food properties it is also important to monitor their compositions; this is especially the case when they contain known food allergens. Globally, there is an increase in the number of people with food allergies, and it has been estimated that 4% of the total world population now suffers from food allergies, with an even higher percentage in infants and children (8%) [5]. The consequences of food allergies are sometimes debilitating, such as celiac disease, and can often be life threatening. There are 14 identified major types of food allergens—celery, cereal containing gluten, crustaceans, eggs, fish, lupin, milk, molluscs, mustard, tree nuts, peanuts, soybeans, and sulphur dioxide [6]. It is important to state that allergen compounds are mostly just proteins or proteinaceous materials and can even be found in low concentrations in products such as fresh fruits and vegetables [7,8]. The majority of allergens listed above can be found in powdered form and are used as ingredients in food products. Despite the significant advances in medical treatments for many diseases, there is no cure for food allergies [5]. Although medicines exist to treat the symptoms of allergic reactions [9], the best method for people with allergies is to ensure they do not eat food containing these allergens. The current techniques for detecting the presence of food allergens are all laboratory-based, requiring a significant amount of time from a skilled individual [10]. For better allergen control within food production environments, there is a need for new technologies to identify materials which naturally contain allergens non-invasively in real-time [11]. Manufacturing is experiencing the fourth industrial revolution, which is the advanced use of data and Industrial Digital Technologies (IDT), such as sensors, artificial intelligence, the Industrial Internet of Things (IIoT), and robotics, to optimise manufacturing processes. Although these technologies are beginning to see widespread use within discrete manufacturing sectors (e.g., automotive), their adoption within process manufacturing environments (e.g., food and drink) is lower, due to barriers such as cost and suitability to operate in challenging industrial environments [12].

### 1.3. NIR Technology Applications in the Food Manufacturing

Near-infrared (NIR) spectroscopy is an optical method, which uses light with wavelengths between 780 and 2500 nm, and is a common method for the quality evaluation of food products [13,14]. Examples of its uses within food and drink manufacturing include identifying the chemical composition of fruits [15,16], vegetables [17,18], and meats [19,20]. NIR spectroscopy has been used to assess several quality parameters for various powder food types. Examples include using NIR to assess the geographical origin of flours [21]. In that study, NIR (1100–2000 nm) was used to scan wheat grain and flour samples, and classification models were built using partial least squares discriminant analysis (PLSDA), with prediction accuracies of 90–96%. The authentication of various powder samples has been performed using NIR spectroscopy [22,23,24,25,26]. Cocchi et al. [23] utilised a Vis/NIR system (400–2500 nm) to identify traces of bread wheat flour in durum wheat flour using soft independence modelling of class analogy (SIMCA) classification models. Ghosh et al. [14] used NIR spectroscopy to differentiate between several cereals (wheat, wheat, oats, spelt, rye, rice, and corn), legumes (soy, white chickpea, poppy, sesame, chia, rapeseed, sunflower seed), and peanut powder. Principal component analysis (PCA) was used as an effective unsupervised clustering technique to identify flours from other samples. In that work, PLSDA was then successfully applied to identify peanut samples from pint nut samples. Another study by Haughey et al. [15] explored the application of NIR spectroscopy (833–2632 nm) to determine soya meal adulteration by melamine, a recently reported adulterant in powdered milk, infant formula, and feed products leading to kidney illnesses and failure. Regression models developed using partial least squares regression (PLSR) had determination coefficient values (R^2^) of 89–99%. Albanell et al. [16] used an NIR technique (1100–2500 nm) to detect low gluten concentrations (0–4.5%) in several gluten-free flours. Modified PLSR models yielded R^2^ values of 68.2–96.7% depending on the adulterant concentration. NIR spectroscopy was also used for determining several quality attributes of flours, such as gliadin, gluten, protein, and moisture contents [27,28,29,30]. Badaró et al. [18] studied the feasibility of using a portable NIR sensor (900–1700 nm) to detect the presence of different dietary fibres (psyllium, bamboo, cellulose) added to semolina flour to enhance their nutritional profile. Classification models developed by the SIMCA technique resulted in 100% classification prediction accuracy, while R^2^ values for regression models were as high as 98%. Wesley et al. [19] applied Vis/NIR spectroscopy (400–2500 nm) to estimate the content of gliadin and gluten content in wheat flour. The regression models developed by PLSR had R^2^ values of 78 and 83% for gliadin and gluten, respectively.

### 1.4. Limitations of Online Applications of the NIR Technology

Although NIR technologies have extensive capabilities for measuring various quality parameters of food products in production environments, there are numerous challenges that must be addressed. Ensuring a representative sample of the material to be measured is a critical factor often affecting the features of the acquired spectra and, consequently, the performance of any classification or regression models developed [31]. Moreover, in powder foods, variations in physical properties (e.g., particle size) will significantly affect the chemical information identified by the NIR system [31]. In the case of diffuse reflectance NIR techniques, light penetrates and scatters inside the material before being reflected back and received by the detector [32]. Determining the optimal configuration of the NIR system for rapid and/or online monitoring of quality attributes is not a trivial task and preliminary experiments are required. It is known that the penetration depth of light into biological materials such as fruit tissues decreases exponentially. This penetration depth is also much less in the NIR region (780–2500 nm) than in the visible region (400–780 nm) [33]. Consequently, any factor that affects the penetration depth should be carefully studied in order to obtain the best configuration for diffuse reflectance measurements. The optimal height distance between the sensor and sample and the sensor light intensity are two important parameters that have to be determined experimentally. These factors are extremely important to consider for NIR systems designed to work in industrial manufacturing environments, where there may be limitations to the minimum distance the sensor can be located from the food sample (to avoid contact with the food and potential safety issues). Near-infrared spectroscopy methods are known to acquire large amounts of spectral data, which need to be pre-processed and analysed using data processing methods, often referred to as chemometrics [34]. General steps for using NIR technology are sample preparation, spectral acquisition, spectral pre-processing, followed by the development of classification and/or regression models [33]. It is known that the pre-processing method applied before the classification algorithms significantly affects the performance of the models [35]. Often, noisy or coarse spectra are acquired by the NIR sensors due to environmental effects (e.g., temperature and humidity), which affects the performance of the models [36]. Therefore, the acquired spectra should be pre-processed using mathematical techniques before being utilised in the machine learning models. Common pre-processing techniques include mean centring, Savitzky–Golay, first derivative smoothing, second derivative smoothing, normalisation, and multiplicative scattering correction (MSC) [34,35]. The appropriate pre-processing method is not always consistent and it differs between sample conditions, data acquisition protocol, and surrounding conditions, such as temperature and moisture. In addition, some spectra pre-processing methods may be more suitable for specific classification models and tests must be performed to determine the most suitable pre-processing methods for a specific application.

This work will focus on the development of an NIR spectroscopy method which utilises machine learning models to identify different powdered food samples which naturally contain allergens such as gluten. The work will focus on measurement parameters and data processing methods, which must be considered when performing measurements in industrial environments. The specific parameters studied are the height between the NIR sensor and the food sample, the sensor light intensity, and the spectra pre-processing methods utilised. A host of different powdered food samples will be studied, including flours, spices, and other materials relevant to the food and drink sector, in addition to a range of different classification machine learning methods.

## 2. Materials and Methods

### 2.1. Raw Materials

**Figure 1:**
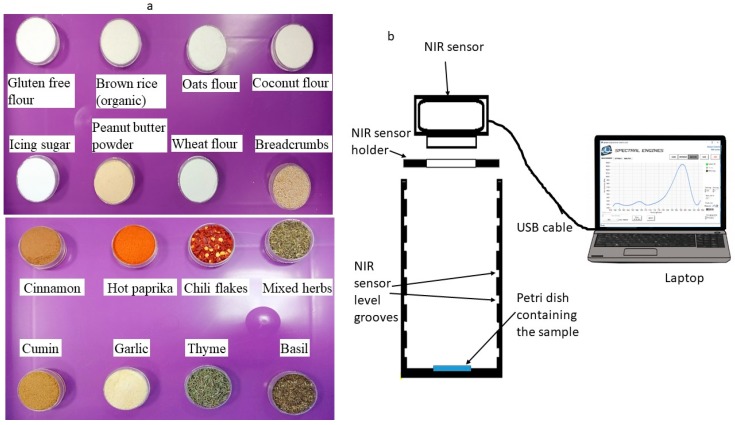
(**a**) Examples of different tested food materials, (**b**) NIR experimental system setup.

In this study, various powdered food materials were tested, some of which naturally contained allergens and others which were naturally allergen-free and not modified after purchasing. All of the samples were purchased from local markets in Nottingham, United Kingdom. A complete list of the materials is available in Table 1. A total of 53 materials were measured in this study. There were 13 gluten-containing flours, 8 gluten-free flours, 7 types of nuts, 4 animal-based powders, and 3 other powdered food materials (salt and sugars). Samples included several different brands of the same powdered food type, to determine if this had any effect of the recorded spectra and classification performance. Each sample was placed inside a 3 cm diameter petri dish. The fine powder samples were compressed by hand using a second empty petri dish to ensure the top surface was flat. Examples of the sample materials in petri dishes are shown in Figure 1a. It should be noted that the tested samples included powders, flakes, and seeds, which have different shapes and would produce differences in the recorded spectra [13,31]. Although some samples could easily be identified by their visible features (colour and shape), this was not possible for many, especially the flours (Figure 1a). The recorded spectral data were divided into two main groups, the Spices Group (SG), which contained 18 different materials, and the Flours Group (FG), which contained flours, nuts, and other non-spice samples. The FG contained 35 different materials. A complete description of the ingredients of each type, adopted from the nutritional labelling, inside the FG is provided in Table 2. Excluding the mixed herbs, which contained thyme, basil, marjoram, and oregano, any material in the SP contained only the named spice, either as a seed or ground to a powder.

### 2.2. Measurement System and Experimental Procedure

The measurement system used for the experiments is shown in Figure 1b. The system comprised a 3D-printed plastic enclosure especially designed for the study. This included slotted groves at different vertical levels (1.5 cm apart), so the height between the food samples and the NIR sensor could be varied. A bespoke holder for the NIR sensor was designed and built to fit into the groves in the plastic enclosure. The NIR sensor used was a NIRONE S2.0, obtained from Spectral Engines, Oulu, Finland. This sensor had a compact shape and light weight (25 × 25 × 17.5 mm^3^, 15 g) making it suitable for integration with manufacturing technologies, such as robots and conveyor systems. The sensor contained two tungsten vacuum lamps with a peak power of 1 W and a single element extended InGaAs detector. The signal-to-noise ratio (SNR) of the sensor was 38.75 dB. The sensor was connected to the laptop via a USB cable. The sensor had a detection range of 1550–1950 nm and a spectral resolution of 1 nm, which made the number of acquired wavelengths 401 for each sample measured. A total of seven different height levels were possible with the holder. However, in this study only six levels were used, as the lowest level was too close to the sample surface to record a stable measurement. In this study, the six levels of sensor heights ranged from 1.5 to 10.5 cm. The light intensities used in the study were 30–90% of the maximum lamp power in 10% increments, resulting in seven levels of light intensities. Consequently, there were 42 possible combinations for height level and light intensity. Each sample was scanned for each combination using five replicates. Before scanning the first sample on a particular day, the sensor was turned on for 10 minutes at a light intensity of 90% to obtain a uniform absorbance. Moreover, at each height level, a reference spectrum was recorded using a white reference disk at 90% light intensity and a dark background measurement at 0% light intensity.

### 2.3. Data Analysis

The relative reflectance for each sample was calculated from the light intensities of the samples (*I_s_*), the reference target (*I_r_*), and the dark (*I_d_*) using the following Equation:

$$Relative\;reflectance=\frac{I_{s}-I_{d}}{I_{r}-I_{d}}.$$

To overcome the effect of noise, either due to electronic sources or natural variations within the samples and the experimental environment, the acquired spectra should be pre-processed [33]. The pre-processing techniques implemented in this study were multiplicative scatter correction (MSC), first derivative smoothing, second derivative smoothing, standard normal variate (SNV), Savitzky–Golay, mean centring, scaling, and robust auto scaling in addition to using the raw spectra without any pre-processing. Such methods are effective in reducing the effect of noise on the spectra quality [37]. Multiplicative scatter correction is designed to reduce the effect of light scattering in spectroscopic data, whereas first and second derivative are smoothing techniques that are mainly applied to minimise the random noise and narrow spikes in spectra [34]. Standard normal variate is based on normalising the rows in the spectra by mean-centring them, then dividing by the standard deviation [34]. Savitzky–Golay is another smoothing method that uses localised polynomial regression to reduce the spikes in the spectra [36]. In this study, the window length for the Savitzky–Golay was chosen as 25. Although multiple different pre-processing methods could be applied to recorded spectra in series, in this work each method was applied individually to understand its effect on classification performance.

Principle component analysis (PCA) is an exploratory data analysis method used for dimension reduction in the case of high dimensional data, where there are more predictors than observations [34,38]. In PCA, the original data is simply transformed into another domain or space, such that the transformed data, usually called scores, are orthogonal [36]. Additionally, PCA also enables better visualisation of the transformed data, making it easier to identify clusters of materials with similar properties based on the recorded spectra. Principle component analysis was performed for each sensor height and light intensity level combination, such that the principal components (PC) could explain at least 99% of the variance between the different powdered food samples. As the number of classes or materials for the FG was relatively high, it was decided to divide the materials into five classes based on either the origin or the main allergen they contain. The first class was gluten-containing materials, and included flours made from wheat, barley, rye, spelt, in addition to oatmeal and wheat gluten. The second class included non-gluten flours, which were flours made from rice, buckwheat, coconut, corn, and tapioca. The third class included animal-based proteins, which were egg white, egg yolk, whole egg, and milk powder. The forth class contained nuts (almond, peanut butter powder, and peanut flour). The fifth class contained caster sugar, icing sugar, and table salt. In the case of the SG, no subgrouping was performed and there were 18 classes, with each class representing one type of spice.

Classification of samples was conducted using several classification machine learning methods, including Linear Discriminant Analysis (LDA), K-nearest neighbour (KNN), SIMCA, PLSDA, and feed forward artificial neural networks (ANN). It should be noted that the classification task was implemented on both the FG (5 classes) and SG (18 classes). Linear discriminant analysis depends on establishing a linear classification threshold and each new object is assigned a class after comparing its discriminant score to the threshold [34]. For the SIMCA method, each class is described by features extracted via PCA such that the PCs for each class are selected irrespective of the PCs for other classes. This enables the optimal feature selection for each sample [34]. The PLSDA technique depends on applying partial least squares regression (PLSR) and assigning the sample to the correct group based on the values of the predicted dependents (y) values, such that the correct class has a predicted value of 1 or −1 [35]. An ANN is a nonlinear, supervised classification technique [34]. The ANN implemented in this study comprised three layers, an input layer that had all pre-processed spectral data, a hidden layer with 50 neurons, and an output layer that contained the sample classes. KNN is a nonlinear classifier that is based on assigning a point to a class whose Euclidean distance to the unknown point is the minimum [34]. The Euclidean distance was chosen in this study to be 4.

For all models, data were divided into a training set (75%) and a test set (25%). To increase the robustness of the deduced classification models, a four-fold cross validation technique was applied only on the training set of data and the optimal classification model obtained was the one that produced the minimum classification error. This optimal model was then used with the test data set. Cross validation is a common technique used when the number of predictors is larger than the number of objects. Thus, the parameters of the obtained models were such that the number of PLS and PCA components were optimised [34].

## 3. Results and Discussion

### 3.1. Effect of Sensor Height on Spectral Signature of Samples

**Figure 2:**
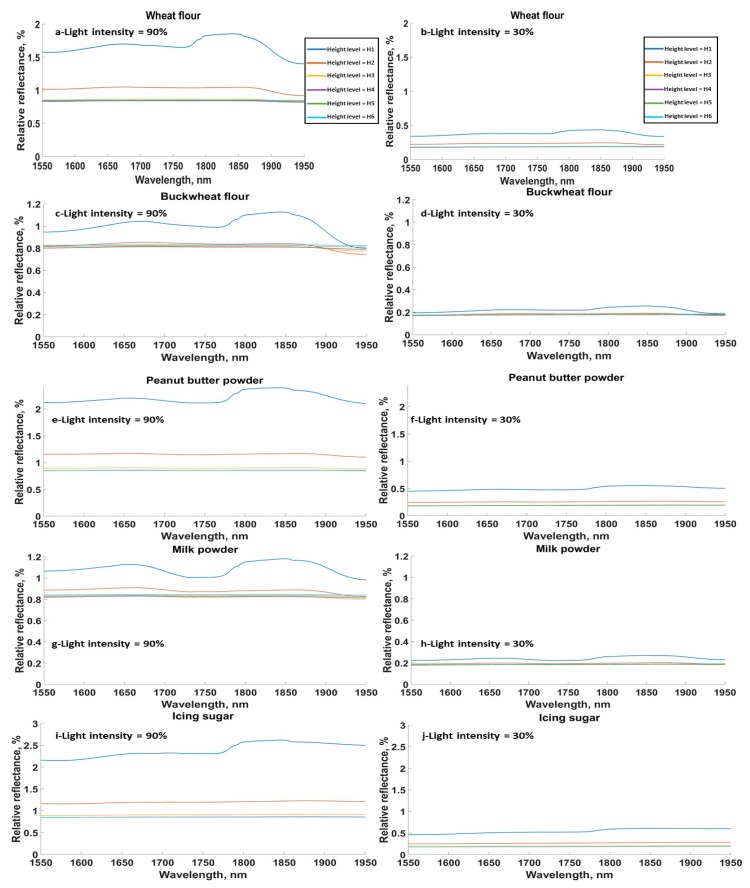
Relative reflectance for all height levels and the minimum and maximum light intensities, 90% and 30%, for (**a**,**b**) wheat flour, (**c**,**d**) buckwheat, (**e**,**f**) peanut butter powder, (**g**,**h**) milk powder, (**i**,**j**) icing sugar. Height levels: H1 = 3 cm, H2 = 4.5 cm, H3 = 6 cm, H4 = 7.5 cm, H5 = 9 cm, H6 = 10.5 cm.

**Figure 3:**
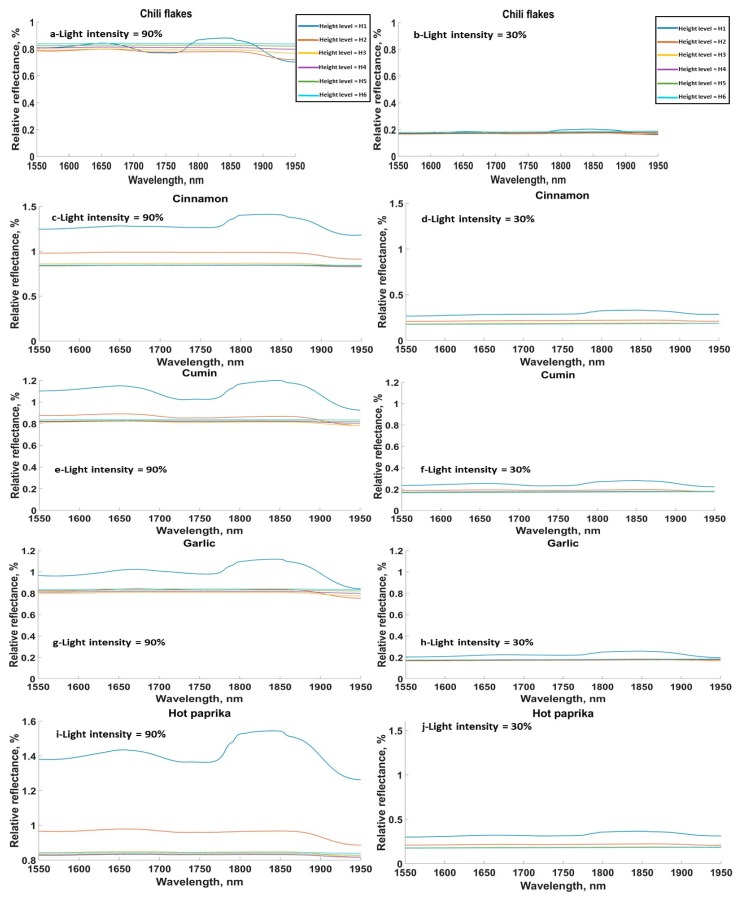
Relative reflectance for all height levels and the minimum and maximum light intensities, 90% and 30%, for (**a**,**b**) chili flakes, (**c**,**d**) cinnamon, (**e**,**f**) cumin, (**g**,**h**) garlic granules, (**i**,**j**) hot paprika. Height levels: H1 = 3 cm, H2 = 4.5 cm, H3 = 6 cm, H4 = 7.5 cm, H5 = 9 cm, H6 = 10.5 cm.

The relative reflectance spectra for different food materials at different sensor heights and light intensities showed a similar trend (Figure 2 and Figure 3). Figure 2a–j shows the relative reflectance spectra for the FG materials, where each sub figure illustrates the relative reflectance at all sensor height levels and one light intensity (either 90% or 30%). Due to the relatively large number of tested materials, only five FG materials are shown, with each one representing one sample from the aforementioned classes. A consistent reduction in the relative reflection was observed around 1780 nm for all samples, especially when the sensor was close to the sample (Levels 1 and 2). This reduction was related to the absorption of water, which was present in all samples [39]. This reduction was not clear for sensor levels beyond H2 (4.5 cm) due to the large level of noise. Additionally, it was found that the intensity of the acquired spectra decreased as either the light intensity reduced or the distance between the sensor and the sample increased.

The SG samples in general showed similar absorption peaks to the FG samples (Figure 3). Relative reflectance spectra are only shown for five materials from the SG in Figure 3 (chili flakes, cinnamon, cumin, and garlic granules). It was found that light intensity drastically affected the amplitude of the acquired spectra, as the relative reflectance spectra at light intensity of 30% was around 20% of the value at the light intensity of 90%. Assuming the air in between the sensor and the sample surface was a uniform gas, then based on Beer-Lambert’s Law, the intensity of the transmitted light decreased exponentially with the path length through the absorbing material.

### 3.2. Effect of Light Intensity on Spectral Signature of Samples

**Figure 4:**
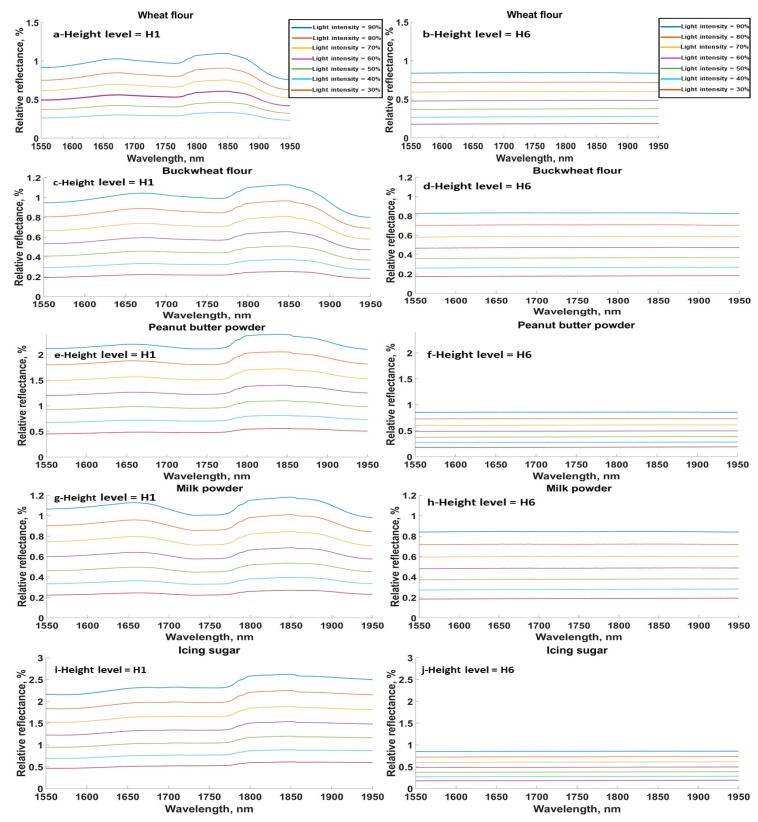
Relative reflectance for all light intensities and the minimum and maximum height levels, H1 and H6, for (**a**,**b**) wheat flour, (**c**,**d**) buckwheat, (**e**,**f**) peanut butter powder, (**g**,**h**) milk powder, (**i**,**j**) icing sugar.

**Figure 5:**
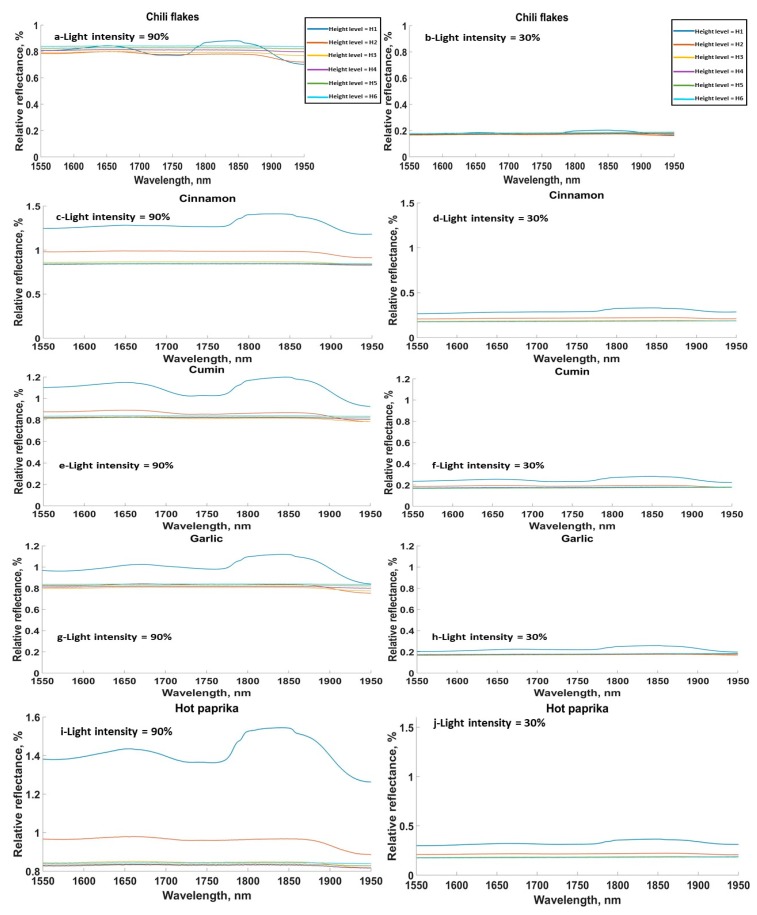
Relative reflectance for all light intensities and the minimum and maximum height levels, H1 and H6, for (**a**,**b**) chili flakes, (**c**,**d**) cinnamon, (**e**,**f**) cumin, (**g**,**h**) garlic granules, (**i**,**j**) hot paprika.

The effect of light intensity on relative reflectance at two height levels, H1 (3 cm) and H6 (10.5 cm), is shown in Figure 4 and Figure 5 for FG and SG, respectively. For consistency, only the spectra for the same powdered food samples as Figure 2 and Figure 3 are shown. It is clear that the moisture absorption peak around 1780 nm had almost disappeared at height H6 for all samples as a result of the more dispersed signals in the surrounding air. However, at heights closer to the sensor, i.e., H1, it was observed that the absorption peaks could still be clearly distinguished for all light intensities. In general, the relative reflectance was proportional to the light intensity for most materials, which follows Beer-Lambert’s law. Generally, the higher the light intensity the more reflected light acquired from samples. Thus, using the sensor at further distances from the sample surface will mostly not help identify some chemical constituents, especially if only low concentrations of the compounds are present.

### 3.3. Effect of Sensor Height and Light Intensity on the Spectra Principle Components

**Figure 6:**
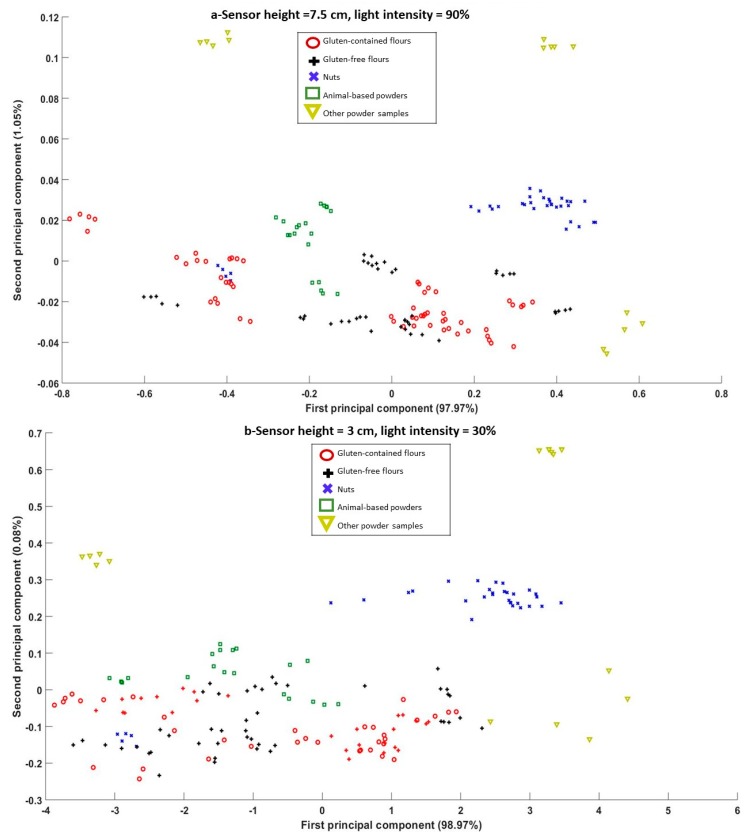
Principal component analysis (PCA) Scatter plots for different flour powder samples scanned by an NIR sensor at (**a**) sensor height level = 7.5 cm and light intensity = 90%, and (**b**) sensor height level = 10.5 cm and light intensity = 30%.

The scatter plots for the PC scores for the five groups of flours samples that illustrated the best and worst class separation are displayed in Figure 6a,b, respectively. For PCA plots it is desirable to have clear separation between different classes so they can be distinguished from each other more easily. The best separation was achieved for height level H4 (7.5 cm) and light intensity 90%, whereas the worst separation was found to be for height level H6 (10.5 cm) and light intensity 30%. It is noted that some materials are well separated in the PCA figures such as icing sugar, caster sugar, and nuts, as a result of the distinguishable composition of such materials. Most nuts contain fatty acids, such as saturated fatty acids, poly saturated fatty acids, and monosaturated fatty acids [40], while the caster or icing sugars solely contain sucrose [41]. However, it was difficult to separate some materials from others at all heights, especially with a higher sensor level or lower light intensity. Moreover, with the highest sensor position from the sample (H6 = 10.5 cm) and the lowest light intensity (30%), the PCs were condensed and represented a situation where identifying the different classes of materials would be challenging. It was found that height level H4 (7.5 cm) along with a light intensity of 90% yielded the best separation of different classes based on the PCA scatter plot (Figure 6a). These results show that different groups were well separated from others, except for almond flour, which was clustered amongst the gluten-containing flours. Two gluten-free flours were also located within the gluten-containing flours cluster. As gluten-free flours mainly contain mixtures of flours of rice, potato, tapioca, maize, and buckwheat, one or more of these individual flours contains common compounds with gluten-containing flours, such as amino acids including phenylalanine, histidine, isoleucine, and leucine [42]. It was found that the effect of light intensity was less important than the sensor height to obtain more separated groups of materials. In a study conducted by Ghosh et al. [24], where several cereals, legumes, nuts, and oilseeds were measured using an NIR sensor (896–1686 nm), the scatter plot of PCs yielded a similar trend, where cereals were well separated from nuts. It is worth stating that with the best combination of height and light intensity shown in Figure 6a, it was possible to separate most allergen-containing foods from foods which did not include allergens, except for the two gluten-free flours discussed above. Thus, it is necessary to also depend upon results obtained from classification models which produce more conclusive results and can be used to quantify the effects of experimental parameters, such as sensor height and light intensity.

**Figure 7:**
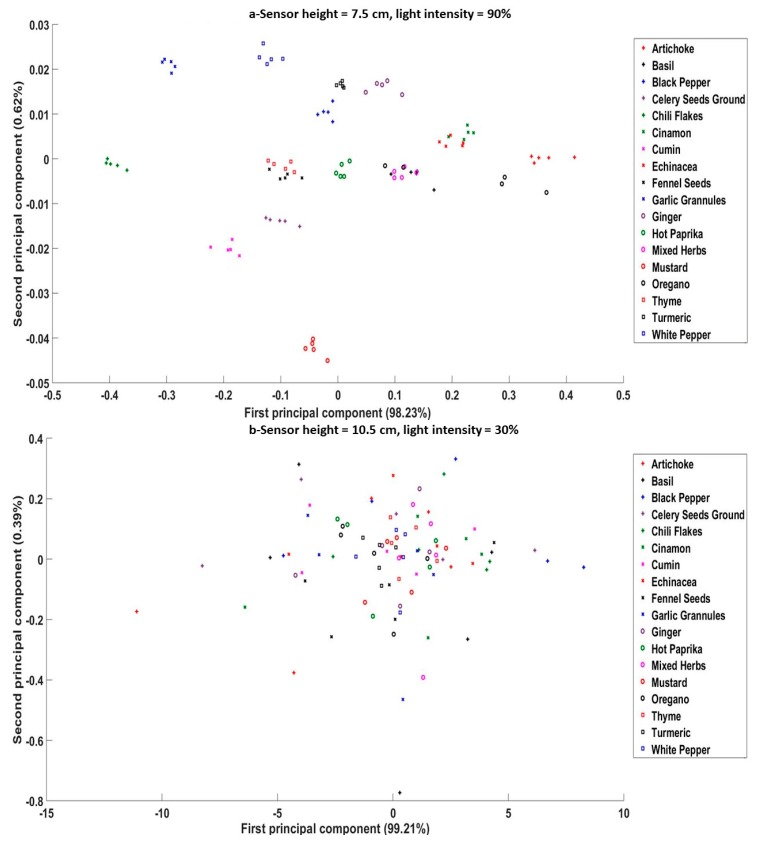
PCA Scatter plots for different spice samples scanned by an NIR sensor at (**a**) sensor height level = 7.5 cm and light intensity = 90%, and (**b**) sensor height level = 10.5 cm and light intensity = 30%.

For the SG, the PCA scatter plots at height levels H4 (7.5 cm) and H6 (10.5 cm), along with light intensities of 90% and 30%, respectively, are presented in Figure 7a,b. The separation of spice samples showed visibly better results than that for flour groups considering the relatively high number of classes (18 for the SG). The light intensity affected the separation more than the sensor height, as can be seen at height levels H1 and H6. Non-separated classes were associated more with light intensities of 30%, with worse results when combining the furthest sensor height with the lowest light intensity. It was clear that samples like mixed herbs, basil, and oregano were clustered together for most height levels, as mixed herbs already contain basil, oregano, and thyme. Celery and mustard were the only materials specified as allergens SG, and both were successfully separated from other samples at the height of 7.5 cm and light intensity of 90%. According to the UK national health service [43], Echinacea triggers allergic reactions for children under the age of 12. In this study, Echinacea was clustered with cinnamon, which illustrates the need to either improve the experimental method (more replicates or wider wavelength range) or combine the PCA results with classification models.

### 3.4. Effect of Sensor Height, Light Intensity, and Pre-Processing Technique on Classification Prediction Accuracy

Classification results for the test data sets of the flour group for all possibilities of sensor height and light intensity combination are shown in Table 3 using KNN, PLSDA, SIMCA, and ANN classification methods. Linear discriminative analysis classification results had much lower classifciation performance and are not displayed here. The clasification prediction accuracies shown are stated as values of minimum–maximum and vary dependeing on the pre-processing technique applied to the spectra. In general, a consistent dependency was found for classification prediction accuracy on sensor height. The further the sensor was from the sample, the more a considerable decrease in classification performance was observed. Classification prediction accuracy also decreased with the lower light intensities at the same sensor height, especially when the sensor was futher from the samples, which matches the results illustrated with the PCA scatter plots (Figure 6 and Figure 7). In addition, KNN generally had the most accurate classification performance over all heights and light intensities studied, and was affected the least by the different pre-processing technqiues. Classification prediction accuracies of up to 100% were obtained for most light intensities for the lowest sensor height (H1 = 3 cm). Furthermore, classification models developed using the KNN method had higher classification performance than other modelling methods for all sensor height and light intensity combinations. The reason KNN resulted in the best classification perormance can be attributed to its simplicity and suitability for multiclass problems [34]. The effect of pre-processing method was more obvious on further sensor heights, where the classification accuracy range was larger. Classification results from the ANNs had fairly consistent values for classification prediction accuracies, although they had the lowest accuracy of all models presented in Table 3. This may be a result of the slightly lower number of samples per class compared with the number of features [44]. In a study conducted by Nawi et al. [44], several pre-processing techniques were used on three different datasets before applying an ANN, and the classification accuracies changed by only a small amount (94.32–99.87%). PLSDA and SIMCA showed similar classification results that decreased to less than 10% prediction accuracy with sensor heights greater than 3 cm. In addition, the different pre-processing methods had significant effects, at all light intensity and height combinations. Classification results obtained in this study are comparable to those by previous studies. Badaró et al. [28] achieved model prediction accuracies of up to 100% for detecting the presence of different dietary fibres added to semolina flour using NIR. Ziegler et al. [45] illustrated that NIR can be used for differetiating between different flours, such as bread, durum, and spelt, with prediction accuracies up to 100%. Althougth the classification results presented in this current work are positive and higlight the potential of the techniques, further work is still required to investigate the effects of other parameters, such as sample particle size and moisture content and enviromental conditions (e.g., temeprature).

The classification prediction accuracy results for spices at different sensor heights and light intensity combinations are shown in Table 4. Results from the ANN models are not shown, as they had poor classification performance. This was possibly due to the relatively large number of classes compared with the number of samples, thus not enabling the effective tuning of network parameters (weights) required to achieve effective models [38]. Linear discriminative analysis and KNN methods yielded the highest classification prediction accuracies. Again, the classification performance decreased with increased sensor height, especially for the measurements at lower light intensities. For sensor heights H1 and H2 (3 cm and 4.5 cm), samples in general were classified correctly (100%), irrespective of light intensity. Starting from sensor height 6 cm, classification prediction accuracies decreased with a consistent reduction with lower light intensities. The classification prediction accuracies were found to be similar when comparing the results from the FG and the SG. Classification results obtained for spices were similar to those available in other published work using spectroscopic measurements. Hu et al. [46] used FT-MIR (4000–40,000 cm^−1^) to assess the adulteration of black pepper by sorghum or Sichuan pepper, with reported classification prediction accuracies between 98–100%. Anibal et al. [47] used a UV/Vis (260–600 nm) to detect Sudan dyes in turmeric, curry, mild paprika, and hot paprika, with resulting classification prediction accuracies up to 99.3%. It is worth stating that PLSDA was generally more affected by the pre-processing techniques than LDA or KNN, and showed considerably reduced classification prediction accuracy when the sensor height was greater than 3 cm. PLSDA mainly depends on using PLS to assign each sample to a class, and thus suffers from the negative effect of small sample sizes when trying to obtain the optimal latent variables. Additionally, PLSDA tends to produce lower prediction accuracy in multiclass problems than binary classification [34]. An example of the confusion matrix obtained from the classification analysis is shown in Table 5. It shows that all groups were classified correctly, except the gluten-containing group, which had 1 sample out of 12 classified as gluten-free. The results illustrate the need for a larger number of samples to improve the classification prediction accuracies. Due to the large number of classes for the spice group, the confusion matrix is not shown.

The pre-processing methods applied to the recorded NIR spectra in this study played a significant role in the performance of the classification models. Amongst all applied pre-processing techniques, SNV and MSC contributed to better classification performance for both FG and SG. Other methods showed slightly lower prediction performance, including Savitzky–Golay, mean centring, and robust auto centring. However, first and second derivatives resulted in the lowest classification prediction accuracy values for the FG and SG. Only the values of classification prediction accuracy for SNV and the second derivative are shown in Table 6, as these methods had the most extreme effects on the classification results. For the models deduced from either technique, it is clear that the prediction accuracy generally decreased the further the sensor was from the sample or with a lower light intensity at the same height level, which agrees with the general results obtained from Table 3 and Table 4. Additionally, it is shown that the data treated with SNV were less affected by the sensor height than those treated with the second derivative. SNV provides a sample-by-sample treatment such that the data will have a zero mean and unit variance, which provides a baseline correction to overcome the problem of having artefacts or noise in the spectra due to any surrounding or uncontrolled conditions produced during measurements [34,35,48]. A possible reason for such low performance for derivative smoothing is the fact that although differentiation might lead to noise removal by minimizing narrow spikes, it might also increase noise that has not presented in the actual spectra [34].

## 4. Conclusions

This work investigated the use of NIR spectroscopy and machine learning models to identify different powdered food materials which naturally contain allergens, such as gluten. The sensor utilised was small, low cost and had a low power requirement, making it suitable for use within production environments. This work specifically focussed on some crucial measurement parameters which ought to be considered when using NIR in real-world environments. These were the height between the sensor and the sample, the light intensity of the sensor, and the pre-processing techniques applied to the recorded spectra. Measurements were made on a range of different powdered food samples, which were split into two main groups, flours and spices. The work showed that using features extracted from the recorded spectra with classification machine learning methods was a suitable approach to identify materials containing allergens, with classification prediction accuracy as high as 100%. It was found that placing the sensor further than 7.5 cm away and using an illumination power less than 70% resulted in poor classification performance. This highlights that users should always identify the most suitable measurement conditions when using these sensors in the laboratory or factory. The study also concluded that amongst the different machine learning methods utilised, KNN and LDA yielded a stable performance of classification prediction accuracy under the studied conditions. Moreover, pre-processing techniques had a significant effect on the classification performance. Methods such as SNV, MSC, and Savitzky–Golay consistently resulted in a more than 100% increase in classification prediction accuracy compared with methods like first and second derivatives. Measurements from the NIR sensor under the optimal combination of sensor height and light intensity resulted in 100% classification prediction accuracy using KNN and LDA methods. However, future work needs to be conducted to cover additional factors that would affect the measured spectra and classification performance. Such factors include the effect of particle size, moisture content, and compositional variations in materials, which may vary between brands. Additionally, to develop measurement systems suitable for real-time data collection within production environments, the ability to perform measurements on moving materials is desirable. Finally, work should be performed to determine the capability of the technique to identify powdered materials adulterated with small quantities of other materials.

## Figures and Tables

**Table 1:** Materials measured by the near-infrared (NIR) sensor.

Flours Group (FG)					Spices Group (SG)
Gluten-Containing Flours	Non-Gluten-Containing Flours	Nuts	Animal-Based Flours	Other	
Barley flour Breadcrumbs Oatmeal Oats flour Rye flour Spelt flour Wheat flour (six brands) Wheat gluten	Buckwheat flour Brown rice flour Brown rice flour (organic) Coconut Corn flour Gluten free flour (two brands) Tapioca flour (organic)	Almond ground Peanut butter powder (four brands) Peanut flour (two brands)	Egg white Egg yolk Egg whole Powdered milk	Casting sugar Icing sugar Table salt	Artichoke powder Basil flakes Black pepper ground Celery ground Chili flakes Cinnamon ground Cumin ground Echinacea powder Fennel seeds Garlic powder Ginger ground Mixed herbs flakes Hot paprika ground Mustard powder Oregano flakes Thyme flakes Turmeric powder White pepper ground
13	8	7	4	3	18

**Table 2:** Ingredients of the materials for the flour group (FG) scanned by the NIR sensor.

Material	Ingredients
Barley Flour	100% barely flour
Breadcrumbs	Wheat flour, salt, yeast
Oatmeal	Wholegrain rolled oats
Oats Flour	Wholegrain oat flour
Rye Flour	Rye flour (organic)
Spelt Flour	Spelt flour
Wheat Flour (Brands 1–3)	Wheat flour, calcium carbonate, iron, niacin, thiamine
Wheat Flour (Brand 4)	Wheat flour, calcium carbonate, iron, niacin, thiamine + ascorbic acid
Wheat Flour (Brand 5)	Wheat flour, calcium carbonate, iron, niacin, thiamine
Wheat Flour (Brand 5)	Wheat flour, calcium carbonate, iron, niacin, thiamine, raising agents: sodium carbonates, calcium phosphates
Wheat Gluten	Powder wheat gluten
Buckwheat Flour	100% buckwheat flour
Brown Rice Flour (Organic)	100% rice flour
Coconut	Organic coconut flour
Corn Flour	Cornflour
Gluten Free Flour	Rice, potato, tapioca, maize, buckwheat, raising agent (mono-calcium phosphate, sodium bicarbonate, thickener (xanthan gum)
Tapioca Flour (Organic)	Tapioca flour
Almond Ground	Almond ground
Peanut Butter Powder (Brands 1 and 2)	Peanut, coconut palm sugar, salt
Peanut Butter Powder (Brand 3)	Peanut flour, Palmyra nectar powder, mineral sea salt
Peanut Butter Powder (Brand 4)	Roasted peanut, sugar, salt
Peanut Flour	100% peanut flour
Egg White	100% pure egg white powder
Egg Yolk	99.7% Egg yolk powder, 0.3% anti-caking agent
Egg Whole	100% pure powdered whole egg
Powdered Milk	Whole milk powder, emulsifier (soya lecithin)
Casting Sugar	Cane sugar
Table Salt	Salt, anticaking agent: sodium ferrocyanide
Icing Sugar	Cane sugar powder

**Table 3:** Classification prediction accuracy for flour and powder samples at different sensor heights and light intensities using different pre-processing methods on the recorded spectra.

	Light Intensity (%)	Classification Range for the Test Set (Minimum–Maximum) (%)			
		KNN	PLSDA	SIMCA	ANN
**Height Level H1**	**90**	77–97	34–77	34–77	51–54
	**80**	71–100	37–69	37–69	51–54
	**70**	71–100	26–83	37–80	54–57
	**60**	77–100	23–83	31–74	54
	**50**	77–100	17–80	31–80	54
	**40**	80–100	17–74	34–74	37–54
	**30**	74–100	6–66	6–66	43–57
**Height Level H2**	**90**	71–97	20–63	3–60	46–54
	**80**	63–97	6–60	6–69	49–54
	**70**	77–100	6–69	6–69	54
	**60**	51–97	9–74	9–74	34–54
	**50**	54–100	3–54	3–54	51–54
	**40**	51–97	6–57	6–57	34–54
	**30**	34–89	3–49	3–46	43–54
**Height Level H3**	**90**	46–94	6–54	6–54	43–54
	**80**	40–91	11–57	11–60	37–54
	**70**	40–94	6–57	6–57	34–54
	**60**	51–89	6–57	6–57	34–54
	**50**	31–86	6–49	6–49	34–54
	**40**	31–83	6–54	6–54	34–54
	**30**	31–94	3–46	3–46	14–60
**Height Level H4**	**90**	49–97	6–46	6–54	43–54
	**80**	34–94	3–37	3–40	43–54
	**70**	31–91	6–51	6–51	37–54
	**60**	29–89	6–43	3–49	40–54
	**50**	26–94	3–34	3–34	31–54
	**40**	49–83	3–43	3–43	34–54
	**30**	23–83	3–46	3–40	34–54
**Height Level H5**	**90**	17–83	6–49	6–46	34–54
	**80**	26–83	3–46	3–40	34–51
	**70**	29–89	6–37	6–46	26–54
	**60**	29–80	3–40	6–40	34–54
	**50**	40–89	3–46	3–46	26–51
	**40**	23–94	3–37	3–37	34–51
	**30**	20–83	6–31	6–31	31–54
**Height Level H6**	**90**	20–89	6–40	6–40	34–54
	**80**	31–83	9–40	9–40	34–54
	**70**	26–77	0–40	0–40	31–54
	**60**	26–69	0–34	0–34	34–60
	**50**	29–74	0–31	0–31	29–57
	**40**	17–69	6–46	6–46	23–54
	**30**	23–66	0–23	0–29	26–43

**Table 4:** Range of classification prediction accuracies for spice samples at different sensor heights and light intensities using different methodologies after scanned using an NIR sensor.

	Light Intensity (%)	Classification Range for the Test Set (Minimum–Maximum) (%)			
		LDA	KNN	PLSDA	ANN
**Height Level H1**	**90**	39–100	44–89	50–89	<20
	**80**	39–100	44–89	61–89	<20
	**70**	39–100	50–100	50–83	<20
	**60**	39–100	50–100	56–94	<20
	**50**	44–100	44–94	39–83	<20
	**40**	50–100	56–100	22–83	<20
	**30**	61–100	50–94	28–72	<20
**Height Level H2**	**90**	61–100	61–100	11–44	<20
	**80**	61–100	61–100	11–67	<20
	**70**	56–100	22–94	17–44	<20
	**60**	33–100	6–94	6–39	<20
	**50**	33–89	11–89	6–39	<20
	**40**	39–100	44–94	6–28	<20
	**30**	44–100	0–72	6–44	<20
**Height Level H3**	**90**	28–94	11–94	6–61	<20
	**80**	39–94	17–94	11–56	<20
	**70**	28–94	11–83	0–17	<20
	**60**	17–94	6–78	6–22	<20
	**50**	11–78	6–72	6–33	<20
	**40**	6–56	6–72	0–22	<20
	**30**	11–67	11–67	0–22	<20
**Height Level H4**	**90**	44–89	6–83	6–22	<20
	**80**	50–78	28–83	6–22	<20
	**70**	56–72	11–83	0–22	<20
	**60**	33–72	17–83	0–17	<20
	**50**	33–67	17–78	0–11	<20
	**40**	28–61	6–78	0–17	<20
	**30**	6–44	0–50	0–6	<20
**Height Level H5**	**90**	17–67	11–61	0–22	<20
	**80**	17–61	6–61	0–28	<20
	**70**	11–72	11–67	0–6	<20
	**60**	22–44	6–61	0–17	<20
	**50**	6–44	0–50	0–6	<20
	**40**	11–33	11–39	0–17	<20
	**30**	0–28	0–33	0–6	<20
**Height Level H6**	**90**	22–72	11–78	6–17	<20
	**80**	11–61	6–72	0–22	<20
	**70**	17–61	0–56	0–11	<20
	**60**	17–50	11–56	0–6	<20
	**50**	0–50	0–67	0–6	<20
	**40**	6–50	6–56	0	<20
	**30**	0–56	0–56	0	<20

**Table 5:** Confusion matrix for classifying different tested materials in the Flour Group (FG) using the NIR sensor and K-nearest neighbour (KNN) classifier with standard normal variate (SNV) pre-processing.

	Gluten-Containing Flours	Gluten-Free Flours	Nuts	Animal-Based Powders	Other Powder Samples	Overall Classification
**Gluten-Containing Flours**	11	1	0	0	0	
**Gluten-Free Flours**	0	9	0	0	0	
**Nuts**	0	0	7	0	0	
**Animal-Based Powders**	0	0	0	4	0	
**Other Powder Samples**	0	0	0	0	3	
						**94%**

**Table 6:** Maximum classification prediction accuracies for flour samples using KNN and spice samples using Linear Discriminant Analysis (LDA) at different sensor heights and light intensities using SNV and second derivative pre-processing on the recorded NIR sprectra.

	Light Intensity (%)	Maximum Classification Range for the Test Set (%)			
		Flours Group Using KNN		Spices Group Using LDA	
		SNV	Second Derivative	SNV	Second Derivative
**Height Level H1**	**90**	97	94	83	100
	**80**	100	100	83	100
	**70**	100	91	78	100
	**60**	100	94	78	100
	**50**	100	91	78	94
	**40**	97	89	78	89
	**30**	100	77	78	83
**Height Level H2**	**90**	97	71	100	50
	**80**	97	63	100	61
	**70**	100	69	100	56
	**60**	97	51	94	33
	**50**	97	54	89	33
	**40**	97	51	100	39
	**30**	83	34	100	44
**Height Level H3**	**90**	94	46	94	28
	**80**	89	40	94	39
	**70**	91	40	94	28
	**60**	77	51	94	17
	**50**	71	31	78	11
	**40**	74	31	56	06
	**30**	74	31	67	06
**Height Level H4**	**90**	83	49	78	44
	**80**	80	34	78	50
	**70**	86	31	61	56
	**60**	69	29	56	33
	**50**	51	26	33	39
	**40**	57	49	33	44
	**30**	49	23	06	11
**Height Level H5**	**90**	63	17	56	28
	**80**	69	26	44	17
	**70**	66	29	33	11
	**60**	46	40	39	22
	**50**	43	40	6	28
	**40**	43	23	11	17
	**30**	34	20	11	0
**Height Level H6**	**90**	57	20	50	22
	**80**	40	31	22	17
	**70**	46	26	17	17
	**60**	31	34	22	22
	**50**	31	29	33	06
	**40**	31	20	6	17
	**30**	31	23	0	6
